# Brood presence and stored food do not modulate forager responsiveness to hexane extracts of insect carcasses in *Temnothorax* ants

**DOI:** 10.17912/micropub.biology.002281

**Published:** 2026-10-21

**Authors:** Laiba Ali, Victoria Patterson, Jaha Hughes, Philip Kohlmeier

**Affiliations:** 1 University of Memphis, Memphis, TN, United States

## Abstract

In social insects, colony nutritional demands modulate foraging, but the underlying sensory mechanisms remain unclear. We tested whether brood presence and stored protein food alter the responsiveness of *Temnothorax longispinosus* ant foragers to chemical extracts of dead flies. Colonies were assigned to treatments varying brood and food cues, and foragers were tested in behavioral choice assays. Neither brood nor stored food altered workers’ preference for fly extracts suggesting that changes in protein-foraging preferences are not mediated by altered individual responsiveness to the hexane-soluble fraction of dead flies, and that recognition of carcasses as protein resources may depend on additional cues.

**Figure 1. Brood and stored food cues do not alter forager responsiveness to Drosophila extracts.** Colonies were maintained without brood and food, with brood but without food, or with brood and food. ( **A** ) Difference in the number of interactions with extract-treated and control filter papers. ( **B** ) Proportion of observations spent on the extract side of the arena. Points represent individual workers; black symbols and error bars show means ± SE. Numbers above groups indicate sample sizes. Asterisks indicate a significant difference from the null expectation ( *p* < 0.05, one-sample t-test); n.s. indicates no significant difference from the null expectation ( *p* ≥ 0.05). ( **C** ) Schematic view of the test arenas used in this study. Panel C was created using Biorender.

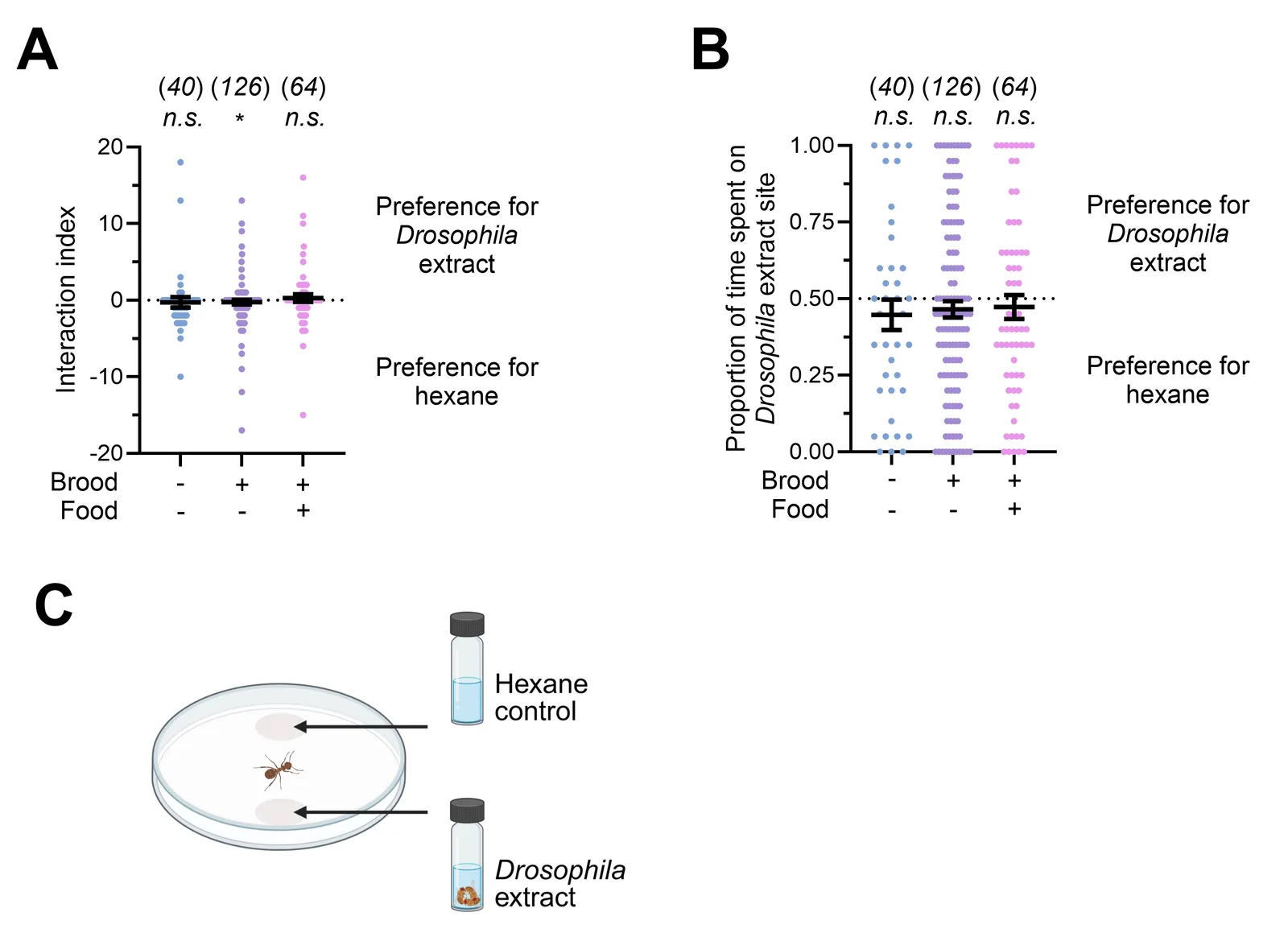



## Description

Animals forage to gather resources required for development, maintenance, and reproduction (Branch and Shen 2017). However, because foraging is energetically costly and exposes animals to risks such as injury and predation, many species have evolved mechanisms to regulate foraging activity in response to physiological demands, allowing them to prevent unnecessary or excessive foraging (Abbott et al. 2026). In insects that do not live in stable social groups and forage only for themselves, this regulation occurs primarily on a physiological and chemosensory level. In the fruit fly *Drosophila melanogaster* , hunger induces temporary and reversible changes in locomotor activity and the chemosensory perception of nutrients (Rajan and Perrimon 2013, Inagaki et al. 2014, LeDue et al. 2016, Branch and Shen 2017) while postingestive feedback from the gut suppresses foraging (Al-Anzi et al. 2010, Branch and Shen 2017).

In social hymenopterans, such as ants, some bees and wasps, only a small proportion of the individuals sharing a nest contribute to foraging (Kwapich and Tschinkel 2013, Kohlmeier et al. 2017, 2018). These foragers have to collect resources for all members of the nest including those that do not forage and those whose nutritional demands do not overlap with those of the foragers. For instance, in many ants, foragers primarily rely on carbohydrates whereas larvae require large amounts of protein (Buschinger and Schaefer 2006, Kohlmeier et al. 2019). In *Temnothorax longispinosus* , a species with behaviorally specialized foragers (Kohlmeier et al. 2017, 2018, 2023), larval chemical cues inside the nest can trigger protein foraging (Dunn et al. 2024). Those foragers will then collect protein i.e., dead insects, and deposit it inside the nest until protein food begins accumulating inside the nest (Abbott et al. 2026). The presence of stored excess food inside the nest then reduces protein foraging (Abbott et al. 2026). In workers of social hymenopterans, task specialization and switches between tasks have been linked to changes in how responsive workers are to task-related cues (Bonabeau et al. 1998). In *T. longispinosus* , a behavioral switch from caring for brood to caring for adult nestmates is associated with a decreased responsiveness towards chemical profiles of brood and an increased behavioral responsiveness towards the chemical profiles of adult nestmates (Kohlmeier et al. 2018).

In this study, we used a combination of nest manipulations and behavioral responsiveness assays in *T. longispinosus* to test whether changes in foraging preferences observed on the colony-level (Dunn et al. 2024, Abbott et al. 2026) are associated with changes in the behavioral responsiveness to chemical cues of dead insects in individual workers. Since protein foraging is upregulated by larval presence (Dunn et al. 2024) and downregulated by cues of food accumulating inside the nest (Abbott et al. 2026), three treatment groups that systematically vary the presence of brood and food cues were created. Colonies were either kept (i) with brood and received ten crushed flies into the nest on the day of the experiment, (ii) with brood but did not receive crushed flies before the experiment, and (iii) without brood and did not receive crushed flies on the day of the experiment. These treatments resulted in colonies in which foragers were exposed to (i) brood and food cues, (ii) brood but not food cues, and (iii) no brood and no food cues. Three hours after these manipulations, foragers were individually transferred to petri dishes and offered two filter papers: one covered with hexane extracts of dead *Drosophila melanogaster* flies and one covered with the hexane solvent alone. If brood cues increase the behavioral responsiveness towards chemical cues of *D. melanogaster* , we predicted that foragers that were exposed to brood but not food cues display a behavioral preference towards the filter paper covered with *D. melanogaster* extracts compared to foragers from the other two groups in which protein foraging was suppressed.

The Interaction index which describes a preference for the *Drosophila* extract-treated disc (if positive) or a preference for the hexane control disc (if negative) was not impacted by treatment group (GLMM: χ ^2^ = 0.93; *p* = 0.628; Figure 1A ). Workers from colonies kept with brood but without food displayed a small preference (interaction index = -0.254 ± 0.312) for the hexane sample (One sample t-test: t = 2.4; *p* = 0.017). The magnitude of this effect corresponds to approximately one additional interaction with the hexane-treated disc per four assays. In contrast, workers from the other treatments showed no detectable preference (Without brood/without food: One sample t-test: t = 1.4; *p* = 0.258. With brood/with food: One sample t-test: t = 0.4; *p* = 0.669). Foragers from colonies with brood but without food cues did not spend more time near the filter paper covered with *D. melanogaster* extracts than foragers from other treatments (GLMM: χ ^2^ = 0.2; *p* = 0.920; Figure 1B ). In none of the treatments did workers spend more time on one of the two sides than on the other (One sample t-tests: t < 1.3; p > 0.203).

Although brood and food cues are sufficient in either triggering or decreasing protein foraging (Dunn et al. 2024, Abbott et al. 2026), we found no evidence that these cues stimulate these behavioral changes through alterations in the behavioral responsiveness to hexane-soluble fractions of the chemical profiles of dead *D. melanogaster* . *Temnothorax longispinosus* workers readily accept intact, dead *D. melanogaster* flies as a food source (Dunn et al. 2024, Abbott et al. 2026) but in the present study did not show attraction toward hexane extracts of flies relative to the solvent control. One possible explanation is that the extract contained insufficient amounts of behaviorally relevant compounds to elicit a response. Published quantitative analyses of individually extracted *D. melanogaster* females report 1.57–1.74 µg of quantified cuticular hydrocarbons per fly, depending on mating status and excluding the male-derived pheromone cVA (Laturney and Billeter 2016). Thus, assuming comparable CHC abundance in the flies used here, the three-fly-equivalent stimulus applied in our behavioral assays would contain approximately 4.7–5.2 µg of CHCs. In comparison, hexane extracts of individual *T. longispinosus* workers from the same population contain approximately 0.399 µg of CHCs, and workers have previously been shown to behaviorally discriminate chemical extracts from single conspecific workers (Kohlmeier et al. 2018). The stimulus used here therefore contained an estimated 11.8–13.1-fold greater total CHC mass than a single-worker extract that is sufficient to elicit behavioral discrimination in this species. Consequently, an insufficient overall quantity of hexane-soluble surface chemicals appears unlikely to fully explain the absence of attraction to the *Drosophila* extract. Another possible explanation is that the hexane-soluble fractions of the surface compounds, including cuticular hydrocarbons, may not be sufficient for workers to identify a dead fly as a protein resource. Recognition may instead depend on additional cues associated with an intact carcass, such as other chemical fractions e.g., the decay-related compound oleic acid (Yao et al. 2009, McAfee et al. 2018), contact/gustatory cues, tactile cues, or combinations of those. In honey bees for instance, changes in gustatory, not olfactory responsiveness are linked to task specialization (Scheiner et al. 2017). Since *T. longispinosus* do not actively hunt but ignore live *Drosophila* flies (Personal observation by PK) and willingly consume them once dead, our results identify an important gap in our understanding of how workers recognize insect carcasses as protein resources. By showing that hexane-soluble surface compounds alone are insufficient to elicit attraction, this study narrows the range of candidate cues involved and provides a basis for future experiments testing which chemical fractions, sensory modalities, or combinations of cues mediate carcass recognition and protein-foraging decisions. In addition, to what extent changes in neurotransmitter release and octopaminergic signaling, which are linked to macronutrient specialization in bees (Arenas et al. 2021), are involved in the activation of protein foraging in ants remains to be tested.

## Methods

A total of 67 *Temnothorax longispinosus* colonies with 1.14 ± 0.06 (mean ± standard error of means) queens and 46.7 ± 3.4 workers were collected in May 2026 at the Edmund Niles Huyck Preserve in Rensselaerville, NY, USA. At the laboratory in the University of Memphis, the colonies were transferred to acrylic glass boxes, along with a nest which consisted of 2 glass slides with a plastic inlay in between, and a moist paper ball to maintain humidity. The colonies were provided with honey and *Drosophila* flies ad libitum for at least five days prior to the experiments.

Colonies were randomly assigned to one of three treatment groups. Colonies in the first group named “With brood; with food” retained their brood and were starved for three days. On the experimental day, approximately ten crushed *Drosophila* flies were placed inside each nest, allowing workers to feed the larvae. These colonies were therefore exposed both to larval cues that stimulate protein foraging and to food-associated cues that suppress protein foraging. Colonies in the second group (“With brood; without food”) also retained their brood and were starved for three days but did not receive crushed flies on the experimental day. These colonies were thus exposed to larval cues that stimulate protein foraging but not to food-associated cues that suppress it. In the third group named “No brood; no food” all larvae were removed before colonies were starved for three days and these colonies did not receive crushed flies on the experimental day. These colonies received neither larval cues that stimulate protein foraging nor food-associated cues that suppress it.

Three hours after food was added to the nests of the “With brood; with food” colonies, all colonies were inspected every 10 min, and workers found outside the nest were collected for behavioral responsiveness assays. In *T. longispinosus* , workers can reliably be identified as foragers based on a single observation of them outside the nest, although they may sporadically contribute to other tasks as well (Kohlmeier et al. 2017, 2018, 2019, 2023). To prepare *Drosophila* chemical extracts, 21 randomly selected flies of the *Memphis-7* strain (Mitchell et al. 2025) were frozen at -18°C. On the day of the experiment, flies were defrosted and kept at room temperature for three hours to allow for the initiation of decay. Flies were then placed in a glass vial, 350 μL hexane was added and the vial was vortexed for 3 min. An aliquot corresponding to the extract of three flies (i.e., 50 μl) was pipetted onto a 1 cm-diameter Whatman filter-paper disc, and the hexane was allowed to evaporate for 30 min. For the solvent control, an equivalent volume of pure hexane was applied to a second filter-paper disc and allowed to evaporate under a fume hood for 30 min.

One disc containing the *Drosophila* extract and one solvent-control disc were placed on opposite sides of a plastic Petri dish (diameter: 5 cm) and a single forager was added to the dish ( Figure 1C ). For ten minutes, the number of interactions with each disc (the only type of interaction observed were antennations) was recorded. In addition, the dish was scanned every 30 seconds and the side of the dish on which the ant was found i.e., the site with the extract or control disc, was recorded yielding 20 observations per ant. Arenas were discarded after each experiment and not re-used to avoid chemical cross-contaminations.

For the statistical analysis, the number of interactions with the hexane filter paper disc was subtracted from the number of interactions with the filter paper disc loaded with the *Drosophila* chemical extracts. This variable was then named “Interaction index”. A Generalized Linear Mixed Model (GLMM) including the interaction index as a response variable, treatment group as an explanatory factor and colony ID as a random effect was run. To test for preferences for one of the two sites of the petri dish, a second GLMM including the proportion of observations spent on the chemical extract site as a response variable, treatment group as an explanatory factor, and colony ID as a random effect was run. One One sample-t-tests per treatment and experiment was run to test for preferences for the *Drosophila* extract/hexane solvent within each treatment group. Statistical analyses were run in R v. 4.5.2 including the packages lme4 and car.

## Reagents

**Table:** 

**Reagent**	**Obtained from**
*Drosophila melanogaster Memphis-7* strain	Collected and established by Philip Kohlmeier.
Hexane	FisherSci, H302SK-4
